# Ipilimumab or FOLFOX with Nivolumab and Trastuzumab in previously untreated HER2-positive locally advanced or metastatic EsophagoGastric Adenocarcinoma - the randomized phase 2 INTEGA trial (AIO STO 0217)

**DOI:** 10.1186/s12885-020-06958-3

**Published:** 2020-06-01

**Authors:** Joseph Tintelnot, Eray Goekkurt, Mascha Binder, Peter Thuss-Patience, Sylvie Lorenzen, Jorge Riera Knorrenschild, Albrecht Kretzschmar, Thomas Ettrich, Udo Lindig, Lutz Jacobasch, Daniel Pink, Salah-Eddin Al-Batran, Axel Hinke, Susanna Hegewisch-Becker, Sven Nilsson, Carsten Bokemeyer, Alexander Stein

**Affiliations:** 1 Department of Internal Medicine II (Oncology Center), University Medical Center Hamburg-Eppendorf, Hubertus Wald Tumorzentrum/UCCH, Martinistr. 52, 20246 Hamburg, Germany; 2 Hematology-Oncology Practice Hamburg (HOPE), Hamburg, Germany; 3 University Hospital Halle-Wittenberg, Halle, Germany; 4 Charité, University Medicine Berlin, Berlin, Germany; 5 Rechts der Isar Hospital, Technical University of Munich, Munich, Germany; 6 University Hospital of Giessen and Marburg, Marburg, Germany; 7 MVZ Mitte, Practice for Hematology and Oncology, Leipzig, Germany; 8 University Hospital Ulm, Ulm, Germany; 9 University Hospital Jena, Jena, Germany; 10 Practice of Hematology and Oncology, Dresden, Germany; 11 Helios Clinic Bad Saarow, Bad Saarow, Germany and University Medicine Greifswald, Greifswald, Germany; 12 Krankenhaus Nordwest, UCT University Cancer Center, Frankfurt, Germany; 13 CCRC, Düsseldorf, Germany

**Keywords:** Esophagogastric adenocarcinoma, HER2, Trastuzumab, Nivolumab, Ipilimumab, FOLFOX

## Abstract

**Background:**

Esophagogastric adenocarcinoma (EGA) currently represents a main cause of cancer related death. Despite an intensified treatment for locally advanced or metastatic EGA with a doublet chemotherapy consisting of a platinum compound and a fluoropyrimidine in combination with trastuzumab for HER2-positive disease or in selected cases with docetaxel, survival remains poor. Recently, immune-oncology based strategies relevantly improved the treatment of different solid tumors and showed some promise in late or later stage trials in EGA. Notably, the combination of immunotherapy with trastuzumab to enhance anti-tumor immunity through activation of innate and adaptive immunity was beneficial in preclinical studies or clinical studies in breast cancer.

**Methods:**

The INTEGA study is an open-label, randomized, multicenter, exploratory phase II trial designed to assess clinical performance, safety and tolerability of ipilimumab or 5-FU/folinic acid and oxaliplatin (FOLFOX) in combination with nivolumab and trastuzumab in patients with previously untreated HER2-positive, locally advanced or metastatic EGA. The primary objective is to determine the clinical performance of ipilimumab or FOLFOX in combination with nivolumab and trastuzumab in terms of overall survival. Secondary objectives are safety and tolerability, efficacy in terms of progression-free survival and objective response rate and blood-based signatures (e.g. immune response or suppression of anti-HER2 resistance) that may correlate with treatment response.

**Discussion:**

Recent evidence from the phase II NCT02954536 study (oxaliplatin, capecitabine, trastuzumab and pembrolizumab) suggests the clinical feasibility of combining chemotherapy, trastuzumab and checkpoint inhibition in EGA. However, evidence for a chemotherapy-free regimen is also mounting in HER2-positive disease (NCT02689284) i.e. margetuximab and Pembrolizumab. Both studies excelled with high overall response rates and manageable toxicities. The INTEGA study aims to comparatively assess these results and select a promising new 1st line regimen which then needs to be confirmed in a randomized phase III trial. Further, the translational part of the study might help to better stratify patients and tailor treatment of either arm.

**Trial registration:**

NCT03409848 24.01.2018.

## Background

Gastric cancer (GC) is the third-most common cause of cancer related death (782,000 deaths) worldwide and the fifth-most common cancer (1 million new cases each year) [1]. The established screening and eradication of *Helicobacter pylori* (HP) decreased the incidence of GC over the past decades [2], meanwhile non-HP derived cancers like gastroesophageal junction (GEJ) cancer increased through risk factors such as obesity and gastroesophageal reflux disease [2, 3]. Further, GC is more frequent among males and its incidence increases with age, peaking between 65 and 74 years [3].

So far, the only curative intended treatment option consists of surgical resection with perioperative chemotherapy or neoadjuvant chemoradiation. Unfortunately, roughly half of these patients suffer a relapse or already have metastatic disease at time of diagnosis, thus leaving palliative chemotherapy the remaining therapy option for most patients with EGA at some time point. The addition of chemotherapy to best supportive care (BSC) led to an increase in overall survival (OS) of 6.7 months (hazard ratio (HR) 0.3), whereas an intensified regimen with combination chemotherapy added another month (HR 0.84) under acceptance of increased toxicities [4]. Therefore, a doublet consisting of a platinum compound and a fluoropyrimidine is currently regarded as standard 1st line treatment in patients with unresectable or metastatic esophagogastric adenocarcinoma (EGA) [5].

In order to stratify the heterogeneity of GCs the Cancer Genome Atlas (TCGA) was able to classify GC into 4 molecular subtypes, namely chromosomal instable (CIN, 50% of all gastric cancers), Epstein-Barr virus positive (EBV, 8%), microsatellite instable (MSI, 22%) and genomic stable (GS, 20%) [6]. Still, human epidermal receptor type 2 (HER2) status, a subgroup of the CIN subtype, is currently the only validated molecular marker to influence treatment-selection in the first-line treatment of advanced disease. The monoclonal IgG1 antibody trastuzumab, in combination with capecitabine or 5-FU and cisplatin, significantly improved survival in patients with HER2-positive disease (defined by immunohistochemistry 3+ or 2+ and amplification), by roughly 4 months compared to chemotherapy alone (HR 0.65) [7]. Unfortunately, HER2-positive disease is only seen in 20% of gastric cancers and 30% of esophageal cancers [8].

The overall outcome of esophagogastric cancer, although relevantly improving during the last decades, remains poor with a median progression-free survival (PFS) limited to 6–7 months and a median overall survival limited to less than 15 months with current standard doublet chemotherapy regimen and licensed antibodies (trastuzumab and ramucirumab) [7, 9, 10].

Thus, the development of efficacious and tolerable combination regimen is urgently required particularly in the 1st line treatment for HER2-positive disease. The INTEGA trial will evaluate two immunotherapy strategies in the 1st line HER2-positive EGA.

### Immunotherapy in gastric cancers

A positive correlation between the infiltration by T cells or natural killer cells and survival was observed in GC patients [11, 12]. This was even more pronounced in the molecular subtypes MSI and EBV [13], underscoring the possible function of immunotherapy in GC. Antibodies targeting immune checkpoint molecules PD-1, PD-L1 or CTLA-4 that limit chronic infection and thereby control immune reactions, recently revolutionized the treatment of different solid tumors like melanoma, renal, bladder and lung cancer [14]. In GC, the first randomized trial comparing nivolumab (anti-PD-1) to placebo (Attraction-02) could observe an increase in OS (5.32 vs. 4.14 months, HR 0.63), PFS (1.61 vs. 1.45 months, HR 0.6) and overall response rate (ORR) (11.2% vs. 0%) [15]. Furthermore, Nivolumab was well tolerated with a safety profile similar to the placebo arm. Other evidence for immune checkpoint inhibition in GC comes from single-arm studies in heavily pre-treated patients using pembrolizumab (anti-PD-1, Keynote-059) or nivolumab (CheckMate 032) with response rates of 11 and 12%, respectively [16, 17].

In contrast to these appealing results the phase III KEYNOTE-061 trial (*n* = 592) (pembrolizumab or paclitaxel) in second-line advanced GC [18] and the JAVELIN Gastric 300 phase III trial (avelumab (anti-PD-L1) or irinotecan/paclitaxel) in third-line advanced GC or GEJ cancer [19], did not result in improved overall survival (OS) but showed a more manageable safety profile than chemotherapy. Notably, protocol specified subgroup analysis showed improved OS in patients with combined positive score (CPS) of 10 or greater (HR 0.64), which is defined by the number of total PD-L1 positive cells divided by the number of viable tumor cells multiplied by 100 [20]. This is in line with the data obtained in the KEYNOTE-181 trial in esophageal cancer [21].

Recently, the KEYNOTE-062 could not demonstrate superiority of pembrolizumab added to chemotherapy in 1st line CPS > 1 EGA despite a favorable trend [22]. On the other hand, non-inferiority of pembrolizumab compared to chemotherapy could be shown in this patient population. Thus, the role of immunotherapy in first line EGA, particularly regarding the combination with chemotherapy or not remains to be determined.

Results from the phase III CheckMate 067 study in advanced melanoma suggest that the combination of PD-1 and CTLA-4 receptor blockade may improve antitumor activity [23] through increased INF-γ production, enhanced CD4/CD8 tumor-infiltrating T-effector cells, and decreased intra-tumor T regulatory cells, as compared to either agent alone [24]. In EGA, the CheckMate 032 study also included two cohorts receiving nivolumab (1 mg/kg) plus ipilimumab (anti-CTLA-4, 3 mg/kg) or nivolumab (3 mg/kg) plus ipilimumab (1 mg/kg) combination therapy with an ORR of 24% or 8%, compared to 12% for nivolumab (3 mg/kg) only. More intriguingly, in PD-L1 > 1% expressing tumors ORR reached 40% (4/10) or 23% (3/13), compared to 19% (3/16) in the nivolumab only group [16]. Response came at a cost of increased treatment-related grade 3–4 adverse events (AEs) in 47, 25% or 17%, respectively. Based on these data the nivolumab 1 mg/kg and ipilimumab 3 mg/kg combination was chosen to be developed further in EGA both in an HER2-negative population (CheckMate 649) and in HER2-positive disease in the presented INTEGA trial.

### Increasing the immune reaction by chemotherapy and HER2-targeting

The induction of immunogenic cell death by oxaliplatin or changes in the immune contexture by 5-Fluouracil (5-FU) showed synergistic effects with checkpoint inhibition in different tumor models [25]. Further, the available trials evaluating the combination of chemotherapy with checkpoint inhibitors have shown feasibility of the combination regimen and a safety profile expected for the individual agents [26]. Results from the ATTRACTION 04 and Keynote-059 study cohort 2 recently showed an acceptable tolerability and high efficacy (ORR 57–76%) for the combination of platinum-based chemotherapy and PD-1 inhibitors in 1st line GC treatment [27, 28].

In addition to the inhibition of the HER2-receptor pathway the IgG1 antibody trastuzumab induces innate and adaptive immunity through antibody dependent cytotoxicity (ADCC) in preclinical models and breast cancer [29–31]. Therefore, trastuzumab could further enhance the immune reaction observed by combining chemotherapy with immune checkpoint inhibition in HER2-positive disease.

### Translational work-up

Although therapy with immune checkpoint inhibitors is promising, effects are limited to subgroups of patients and up to date no biomarkers are available to reliably select responding patients. The newly classified molecular subtypes may help to identify responders with subgroups like EBV or MSI to be more immunogenic [32, 33]. In addition, resistance to HER2-targeting in HER2-positive tumors might be present upfront or will eventually develop during treatment, particularly by loss of HER2 amplification [34].

Here we will use liquid biopsy techniques to analyze HER2-receptor status, analyze the repertoire of tumor-infiltrating lymphocytes (TiL) and analyze circulating tumor cells (CTCs) with the ultimate goal to reveal molecular or immunological profiles of responder patients.

### Study objective

The primary objective is to determine the clinical performance of ipilimumab or 5-FU/folinic acid and oxaliplatin (FOLFOX) in combination with nivolumab and trastuzumab in patients with previously untreated HER2-positive locally advanced or metastatic esophagogastric adenocarcinoma in terms of OS.

The main secondary objective is to determine safety and tolerability, according to NCI CTCAE v4.03 and to the obtained data on vital signs, clinical parameters and feasibility of the regimen. Other secondary objectives are to determine efficacy in terms of PFS and ORR rate according to RECIST v1.1 of the experimental regimen. In addition, immune response signatures (e.g. TiL repertoire and next-generation sequencing (NGS) immunoprofiling of immunoglobulins and T-cell receptor rearrangements), changes in HER2 and PD-L1 status in CTCs and ctDNA will be correlated with efficacy.

## Methods/design

**Fig. 1:**
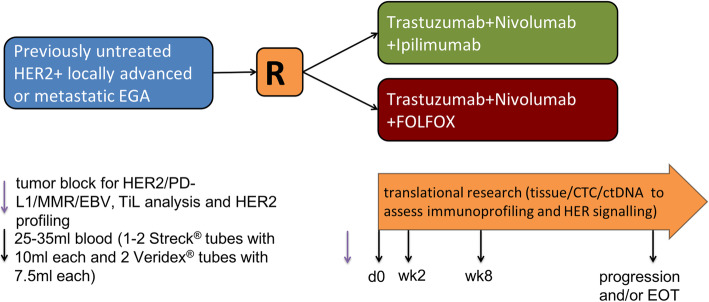
Study schedule overview

The INTEGA study is an open-label, randomized multicenter phase II trial designed to asses clinical performance, safety and tolerability of ipilimumab or FOLFOX in combination with nivolumab and trastuzumab in patients with previously untreated HER2-positive locally advanced or metastatic esophagogastric adenocarcinoma. Ninety-seven patients should be recruited over a duration of 24 months. Follow-up for survival should last 48 months from inclusion of the first patient (Fig. 1). Participating hospitals are located in Germany and listed in Supplementary Table 1.

### Treatment

Eligible patients (Table 1) will be randomized to receive either trastuzumab, nivolumab and ipilimumab (Arm A) or trastuzumab, nivolumab and mFOLFOX (Arm B).

**Table 1:** Inclusion criteria for the INTEGA study

• All subjects must have inoperable, advanced or metastatic esophagogastric adenocarcinoma.	
• Subjects must have HER2-positive disease defined as either IHC 3+ or IHC 2+, the latter in combination with ISH+, as assessed locally on a primary or metastatic tumor (*Note:* Availability of formalin-fixed paraffin-embedded (FFPE) representative tumor tissue for central confirmation of HER2 is mandatory (Preferably fresh biopsy))	
• Subject must be previously untreated with systemic treatment (including HER 2 inhibitors) given as primary therapy for advanced or metastatic disease.	
• Prior adjuvant or neoadjuvant chemotherapy, radiotherapy and/or chemoradiotherapy are permitted as long as the last administration of the last regimen (whichever was given last) occurred at least 3 months prior to randomization.	
• Subjects must have measurable or evaluable non-measurable disease as assessed by the investigator, according to RECIST v1.1.	
• ECOG performance status score of 0 or 1.	
• Screening laboratory values must meet the following criteria (using NCI CTCAE v.4.03):	
o WBC ≥ 2000/uL	
o Neutrophils ≥1500/μL	
o Platelets ≥100 × 10^3^/μL	
o Hemoglobin ≥9.0 g/dL	
o eGFR ≥30 ml/min	
o AST ≤ 3.0 x ULN (or ≤ 5.0X ULN if liver metastases are present)	
o ALT ≤3.0 x ULN (or ≤ 5.0X ULN if liver metastases are present)	
o Total Bilirubin ≤1.5 x ULN (except subjects with Gilbert Syndrome who must have a total bilirubin level of < 3.0 x ULN)	
• Males and Females, ≥ 18 years of age	
• Subjects must have signed and dated an IRB/IEC approved written informed consent form in accordance with regulatory and institutional guidelines. This must be obtained before the performance of any protocol-related procedures that are not part of normal subject care.	
• Subjects must be willing and able to comply with scheduled visits, treatment schedule, laboratory tests and other requirements of the study.	
• Women of childbearing potential (WOCBP) must have a negative serum or urine pregnancy test (minimum sensitivity 25 IU/L or equivalent units of HCG) within 24 h prior to the start of study drug. Women must not be breastfeeding.	
• WOCBP must use a highly effective method(s) of contraception for a period of 30 days (duration of ovulatory cycle) plus the time required for the investigational drug to undergo 5 half-lives. The terminal half-lives of nivolumab and ipilimumab are approximately 25 days and 15 days, respectively. WOCBP should use an adequate method to avoid pregnancy for approximately 5 months (30 days plus the time required for nivolumab to undergo 5 half-lives) after the last dose of investigational drug.	
• Males who are sexually active with WOCBP must agree to follow instructions for method(s) of contraception for a period of 90 days (duration of sperm turnover) plus the time required for the investigational drug to undergo 5 half-lives. The terminal half-lives of nivolumab and ipilimumab are approximately 25 days and 15 days, respectively. Males who are sexually active with WOCBP must continue contraception for approximately 7 months (90 days plus the time required for nivolumab to undergo 5 half-lives) after the last dose of investigational drug. In addition, male subjects must be willing to refrain from sperm donation during this time.	

Randomization will be performed according to the following stratification criteria:

- Prior surgery of the primary tumor (yes vs. no)
- HER2 status immunohistochemistry (IHC) 3+ vs. IHC 2+ and in-situ hybridization (ISH) amplified

All used agents nivolumab, ipilimumab, trastuzumab, 5FU, folinic acid and oxaliplatin will be defined as investigational medicinal product (IMP).

#### Arm A

Patients assigned to arm A will receive trastuzumab 6 mg/kg i.v. every 3 weeks (loading dose 8 mg/kg), nivolumab 1 mg/kg i.v. every 3 weeks and ipilimumab 3 mg/kg i.v. every 3 weeks for a total of 12 weeks. From week 13, patients receive trastuzumab 4 mg/kg i.v. every 2 weeks and nivolumab 240 mg i.v. every 2 weeks.

#### Arm B

Patients assigned to arm B will receive trastuzumab 4 mg/kg i.v. every 2 weeks (loading dose 6 mg/kg), nivolumab 240 mg i.v. every 2 weeks and mFOLFOX6 every 2 weeks (oxaliplatin at a dose of 85 mg/m^2^ i.v. over 2 h (day 1), 5-FU 400 mg/m^2^ i.v. bolus (day 1), folinic acid at a dose of 400 mg/m^2^ i.v. over 2 h (day 1) and 5-FU at a dose of 2400 mg/m^2^ i.v. over 46 h (day 1–3) according to [35]).

Treatment with trastuzumab, nivolumab and ipilimumab or FOLFOX will be administered until progression (according to RECIST v1.1), intolerable toxicity, withdrawal of consent or secondary resection. The treatment with nivolumab will be limited to a maximum of 12 months (24 applications of nivolumab). Ipilimumab will only be applied in weeks 1, 4, 7, and 10.

An independent data monitoring committee (IDMC) will monitor safety data every 3 to 6 months throughout the trial. In addition, a safety run-in phase for the first 15 patients will be conducted to detect potential safety risks early. After at least 2 months of treatment of the 5th, 10th and 15th patient per arm the IDMC will review the safety data respectively and decide about trial continuation.

### Assessments

Baseline assessment is performed within 4 weeks prior treatment (Table 2).

**Table 2:** Baseline assessment

• Review of inclusion and exclusion criteria	
• Medical and medication history, physical examination including height, weight, vital signs (blood pressure, heart rate, respiratory rate, body temperature), oxygen saturation, ECOG-performance status	
• Laboratory Tests:	
o Hematology panel: hemoglobin, platelets, white blood cell (WBC) count and WBC differential (neutrophils, lymphocytes)	
o Chemistry panel: sodium, potassium, calcium, magnesium, creatinine, urea, total bilirubin, alkaline phosphatase, alanine aminotransferase (ALT), aspartate aminotransferase (AST), total protein, albumin, LDH, glucose, amylase, lipase	
o Free T3/T4 and TSH	
o Coagulation: INR, aPTT	
o CA 72–4 (CEA, CA 19–9 optional)	
o Hepatitis B/C screening test (HBsAg, anti-HBc, anti-HBs, anti-HCV)	
o HIV screening test (HIV 1/2 antigen/antibody test)	
o Pregnancy test for women of childbearing potential within 24 h prior to start of the treatment	
• Blood draw for translational research	
• Obtain paraffin-embedded tumor-tissue for translational research	
• Echocardiography and ECG	
• Quality of life assessment (EORTC QLQ-C30 and STO-22)	
• Disease assessment by radiological imaging of the chest, abdomen, pelvis and all other sites of disease (CT/MRI-scan)	

During treatment assessment is done according to Table 3 every 2 or 3 weeks. In addition, arm A further includes an extra assessment on day 12 of every cycle until week 13.

**Table 3:** During treatment assessment

• Physical examination including oxygen saturation, performance status (ECOG), assessment of toxicity, concomitant medication	
• Laboratory tests (hematology and chemistry panel), including	
• Free T3/T4 and TSH (every 6 weeks)	
• Pregnancy test for women of childbearing potential (every 4 weeks)	
• Quality of life assessment (EORTC QLQ-C30 and STO-22) every 2 months (together with imaging)	
• Blood draw for translational research (cycle 2, cycle 4/5 [Arm A/B] and progression and/or end of treatment)	
• Echocardiography every 3 months	
• Disease assessment by radiological imaging of the chest, abdomen, pelvis and all other sites of disease (CT/MRI-scan) every 8 weeks for up to 12 months and thereafter 3 monthly	
• Quality of life will be assessed using the EORTC QLQ-C30 and STO-22 every 8 weeks together with tumor response assessment	
Additional assessments during treatment with nivolumab, ipilimumab and trastuzumab in arm A until week 13 on day 12 of every cycle (+/−3 days)	
• Physical examination including oxygen saturation, performance status (ECOG), assessment of toxicity, concomitant medication	
• Laboratory tests (hematology and chemistry panel)	

During treatment tumor response will be assessed every 8 weeks (±7 days) for up to 12 months and afterwards 3 monthly by CT and/or MRI of the chest, abdomen, pelvis and all other sites of disease. After treatment discontinuation for other than progressive disease imaging will be performed according to standard of care until progression or death. CT and/or MRI scans will be independently reviewed, thus blinded data will be collected.

When any subject discontinues the study treatment, the final assessments should be made according to Table 4.

**Table 4:** Final staging

• Physical examination including oxygen saturation, performance status (ECOG), assessment of toxicity, concomitant medication	
• Laboratory tests (baseline panel), including free T4 and TSH and pregnancy test for women of childbearing potential	
• Echocardiography and ECG	
• Disease assessment by radiological imaging of the chest, abdomen, pelvis and all other sites of disease (CT/MRI-scan)	

### Follow-up

All subjects will be followed every 3 months ±28 days for up to 4 years after start of recruitment (Table 5).

**Table 5:** Follow-up

In case of progressive disease after study treatment only:	
• Survival, disease status, protracted toxicity, further treatment	
In any other case additionally:	
• Disease assessment, physical examination including weight, ECOG-performance status	
• Blood draw for translational research at progression	

Given the potential risk for delayed immune-related toxicities, safety follow-up (Table 6) must be performed every 30 days up to 100 days after the last dose of IMP. The extended safety follow-up beyond 30 days (60 / 100 days) after last study drug administration may be performed either via a site visit or via a telephone call with subsequent site visit requested in case any concerns noted during the telephone call.

**Table 6:** Safety follow-up

• Physical xamination including oxygen saturation performance status (ECOG) assessment of toxicit concomitant medication	
• Laboratory tests (hematology and chemistry panel) including free T3/T4 and TSH and pregnancy test for women of childbearing potential	

### Material collection for translational work

The tumor block for TiL analysis, HER2, PD-L1 and HER2 signaling assessment will be obtained at baseline. Blood will be collected prior to first treatment and at the beginning of cycle 2 and 4/5 (Arm A/B) and at progression and/or end of treatment (Table 7). In addition, imaging will be retrospectively collected.

**Table 7:** Translational work-up

• Tumor-infiltrating lymphocytes (TiL) repertoire determination from tumor	
• Liquid biopsy next-generation sequencing (NGS) immunoprofiling (*TCRβ* & *IgH*) before treatment initiation and before second cycle to determine response predictive immune signature (diversification pattern as read-out for ongoing immune activation, TiL clone expansion in peripheral blood)	
• In addition, FFPE will be centrally tested for PD-L1, HER2 (IHC and ISH), MSI, EBV and HER signaling alterations (amplifications and/or mutations in e.g. EGFR, HER2, HER3, PIK3CA) and correlated with clinical efficacy	
• CTC will be evaluated for changes in HER2 and PD-L1 status	
• ctDNA will be evaluated for HER signaling alterations (amplifications and/or mutations in e.g. EGFR, HER2, HER3, PIK3CA)	
• Central imaging review and determination of ORR and PFS according to modified RECIST	

### Analysis of study endpoints

The study population will be analyzed for the primary endpoint (OS) when 71 events have been observed. When the last patient has passed the 3 months safety assessment after completion of up to 12 months of nivolumab the final safety analyses will be conducted. Further follow up for survival will be performed for overall 4 years (counted from first patient inclusion). The completion of the overall survival follow-up will be the end of the trial.

### Statistical considerations and data handling

The present trial is designed as a randomized phase II study, which aims to estimate the therapeutic efficacy of two experimental regimen. OS analyzed according to the intention-to-treat (ITT) principle is the primary efficacy endpoint. The efficacy assumptions are derived from historical data.

The TOGA trial has defined the standard 1st line treatment with chemotherapy and trastuzumab with a 12-month-OS rate (OSR@12) of 55% (median OS of 13.8 months). Nivolumab in chemotherapy refractory patients (median 3 prior treatment lines) resulted in an overall response rate of 11–14% and a median OS of about 5.3 months. The combination of nivolumab and ipilimumab in the same patient population resulted in an overall response rate of 26% and a median OS of about 6.9 months. The INTEGA trial will evaluate two experimental regimens in 1st line HER2-positive esophagogastric adenocarcinoma treatment, a chemo-free regimen with trastuzumab, nivolumab and ipilimumab and an intensified TOGA-like regimen with trastuzumab, nivolumab and FOLFOX. Each of the experimental arms would be considered promising, if the true 12-month OS rate amounts to 70%. This translates into a hazard ratio of 0.6 compared to the standard OSR@12 of 55% for chemotherapy and trastuzumab.

### Sample size estimation

Based on these assumptions, and an exponential shape of the survival curves, a one-sided log-rank test with a sample size of 41 subjects achieves 80% power at a one-sided significance level of 0.05 to detect a hazard ratio of 0.6 against an assumed fixed OSR@12 of 55% with the current standard. Overall 82 patients will be included and randomized into the two experimental arms (41 in each experimental treatment group). The rate of drop-outs is estimated to be 15%. Hence, the total number of subjects to be recruited is *N* = 97. This calculation assumes an accrual time of 24 months, and a minimum follow-up of 15 months of all patients alive at the time point of analysis.

## Discussion

In metastatic or advanced HER2-positive GC or GEJ cancer fluoropyrimidine, platinum and trastuzumab remains the current standard of care with a limited median overall survival of 14 months [7]. Intensification of the HER2 blockade by adding pertuzumab in the 1st line situation did not improve survival in esophagogastric cancer in contrast to breast cancer [36]. Targeting HER2 was not efficacious in the second line setting as recently shown in the phase 3 GATSBY trial [37]. Therefore, HER2-targeting is clearly confined to the 1st line setting.

Together the development of efficacious and tolerable combination regimen is urgently required particularly in the 1st line treatment for HER2-positive disease. The combination of immunotherapy and HER2-targeting agents is of high interest in EGA. This was recently underlined by two phase II studies. The Fc-modified next generation HER2-antibody margetuximab showed interesting results in trastuzumab refractory patients in combination with pembrolizumab [38]. The ORR was 16% with 54% disease control rate (DCR) with this chemotherapy-free treatment regimen. Especially the group of PD-L1 positive and HER2 amplifying tumors analyzed by ctDNA excelled with an ORR of 57 and 86% DCR. Grade 3–4 adverse events were noted in only 15.6% of patients [38]. The other study tested the combination of pembrolizumab, trastuzumab, capecitabine and oxaliplatin as 1st line treatment of EGA with an extraordinary ORR of 87% and PFS of 11.3 months [39]. Based on these results, the Keynote 811 phase III trial currently evaluates the addition of pembrolizumab with current HER2-positive standard regimen of fluoropyrimidine, platinum and trastuzumab (NCT03615326). Since this combination almost mirrors arm B of the INTEGA trial, it will be interesting to see whether the INTEGA trial is able to reproduce these encouraging results much earlier.

The experimental regimens evaluated in this trial combine the 1st line standard drug of trastuzumab with the PD-1 antibody nivolumab and either the broadly tolerable and efficacious standard regimen FOLFOX or in a completely chemo-free regimen with ipilimumab. Thus, in the FOLFOX, trastuzumab and nivolumab arm patients will receive the current standard regimen of platinum-based chemotherapy with trastuzumab intensified by nivolumab, which may increase efficacy of both the chemotherapy and the HER2 blockade. Based on the currently available data a decrease in efficacy due to the investigational combination of standard 1st line treatment with nivolumab is unlikely. In arm A a chemotherapy-free regimen will be applied. However, arm A contains the proven efficacious trastuzumab, which is a part of the current standard 1st line treatment and the combination of ipilimumab and nivolumab will be applied. Due to the effectivity of the chemotherapy-free checkpoint inhibitor combination (nivolumab combined with ipilimumab) as a salvage therapy in the CheckMate 032 trial or the combination of margetuximab and pembrolizumab, the addition of standard of care trastuzumab and the potential synergistic effect of trastuzumab and checkpoint inhibition, an inferiority compared to the chemotherapy arm is not expected. Nevertheless, to account for potential inferiority of either experimental arms, close meshed CT scans every 8 weeks will be conducted to detect early progression and enable immediate switch to chemotherapy or second line treatment.

Based on the available data on FOLFOX in combination with PD-L1 antibodies and HER1/EGFR antibodies with PD-L1 antibodies demonstrating the feasibility and general tolerability of these two combinations, this phase II trial will start with a full dose of trastuzumab, nivolumab and either ipilimumab (dose of 3 mg/kg for 4 doses once every 3 weeks) or FOLFOX (according to the mFOLFOX6 regimen). Adverse events have been broadly consistent across tumor types following monotherapy and have not demonstrated clear dose-response or exposure-response relationships. In dual checkpoint inhibition, however, increased numbers of AEs have been observed, the equally dosed Checkmate 032 cohort reported 47% grade 3–4 AEs [16]. To carefully evaluate potential critical toxicities patients will be closely monitored including assessments for risk of interstitial lung disease and a continuous safety analysis for every 5th patient per arm passing the 2 months assessment during the safety run-in phase (first 15 patients) and every 3 months thereafter will be conducted.

Regarding the potential AEs and the limited benefit of immunotherapy for some patients, predictive markers to tailor treatment are urgently warranted either at baseline or early during treatment. PD-1 may serve as such biomarker in some tumor subtypes [40]. In GC or GEJ cancer, several studies reported a favorable response in PD-1 expressing subsets [17, 41]. Other studies, however, did not observe any difference in response to checkpoint inhibition tailored by PD-1 expression [18, 19, 39]. Also in terms of HER2-targeting by trastuzumab a molecular characterization is needed since several mechanisms of treatment induced resistance might be present upfront or will eventually develop during treatment, particularly loss of HER2-amplification [34]. The recently published results using HER2-targeting in combination with immunotherapy showed an anticipated benefit for patients expressing HER2 [39] or expressing both PD-1 and HER2 [38]. Here we will assess baseline FFPE and ctDNA for HER2, HER signaling alterations (amplifications and/or mutations in e.g. EGFR, HER2, HER3, PIK3CA), CTCs for HER2 and PD-L1 expression and baseline FFPE for PD-L1, MSI and EBV to validate baseline markers with potential or likely predictive value for checkpoint-inhibition and HER2-targeting, although the coincidence of at least MSI and EBV with HER2-amplification is rare [6]. Immunoprofiling by liquid biopsy will be performed prior to treatment initiation and before the second nivolumab dose to determine response predictive immune signatures since diversification patterns can be exploited to separate responder and non-responder patients in other tumor subtypes like melanoma [40, 42].

In summary, the INTEGA trial may thus establish a new 1st line regimen candidate with potentially increased efficacy and acceptable tolerability, which then needs to be validated compared to current HER2-positive standard regimen of fluoropyrimidine, platinum and trastuzumab in a randomized phase III trial. The analysis of immune profiles and expression data might help to fulfil the urgent need of biomarkers to tailor treatment in this setting of immunotherapy.

## Supplementary information

**Additional file 1.**

## Data Availability

Not applicable.

## Abbreviations

5-FU: 5-Fluouracil
FOLFOX: 5-FU/folinic acid and Oxaliplatin
AEs: Adverse events
ALT: Alanine aminotransferase
ADCC: Antibody dependent cytotoxicity
AST: Aspartate aminotransferase
CIN: Chromosomal instable
CTC: Circulating tumor cells
DCR: Disease control rate
HER2: Epidermal receptor type 2
EBV: Epstein-Barr virus positive
EGA: Esophagogastric adenocarcinoma
FFPE: Formalin-fixed paraffin-embedded
GC: Gastric cancer
GEJC: Gastroesophageal junction cancer
GS: Genomic stable
HR: Hazard ratio
HP: *Helicobacter pylori*
IHC: Immunohistochemistry
IDMC: Independent data monitoring committee
ISH: In-situ hybridization
ITT: Intention-to-treat
MSI: Microsatellite instable
NGS: Next-generation sequencing
ORR: Overall response rate
OS: Overall survival
PFS: Progression-free survival
TiL: Tumor-infiltrating lymphocytes
WBC: White blood cell
WOCBP: Women of childbearing potential
